# Application of Reverse Engineering and Numerical Simulation in the Development of a High-Pressure Die Casting Process

**DOI:** 10.3390/ma19214675

**Published:** 2026-11-03

**Authors:** Karlo Jurković, Tomislav Staroveški, Branko Bauer, Tomislav Gotić

**Affiliations:** 1 Department of Technology, University of Zagreb, 10000 Zagreb, Croatia; tomislav.staroveski@fsb.hr (T.S.); branko.bauer@fsb.hr (B.B.); 2 Fripol d.o.o., 42220 Ljubešćica, Croatia; t.gotic@fripol.com

**Keywords:** high-pressure die casting, reverse engineering, 3D optical scanning, numerical simulation, casting defects

## Abstract

The paper presents the application of 3D optical scanning and numerical simulation in the development of a high-pressure die casting process for a differential transmission cover. The geometry of the existing casting was digitized using a 3D optical scanner, followed by reconstruction of the CAD model using reverse engineering. Based on the reconstructed geometry, the gating system and overflow elements were defined, and a numerical model of the die casting process was developed. Numerical analysis was performed using the ProCAST software package, with the filling of the die cavity, solidification of the molten metal, temperature distribution, and the potential occurrence of characteristic casting defects, including air entrainment and shrinkage porosity, being analyzed. The simulation results showed favorable filling and solidification behavior, with only localized areas exhibiting an increased potential for shrinkage porosity. The conducted research demonstrates the applicability of combining 3D scanning, reverse engineering, and numerical simulation as an effective approach to the development and optimization of die casting processes.

## 1. Introduction

High-pressure die casting (HPDC) is one of the most important casting processes used in the automotive, electrical, and consumer goods industries due to its ability to produce complex geometries with high dimensional accuracy, good surface quality, and short production cycle times [1]. The process is based on injecting molten metal into a metallic die at high pressure and high velocities, enabling the mass production of castings with high process repeatability [2]. Depending on the machine configuration, high-pressure die casting can be classified into hot-chamber and cold-chamber die casting. Hot-chamber machines are used for lower-melting-point alloys, such as zinc and magnesium alloys, where the shot chamber and injection system are immersed in the molten metal, enabling very short production cycles. In contrast, in cold-chamber machines, the molten metal is ladled into the shot sleeve immediately before injection, making them suitable for casting higher melting point alloys, primarily aluminum and copper alloys [3,4]. Aluminum alloys are currently among the most widely used materials in die casting due to their favorable strength to weight ratio, good castability, and suitability for the production of complex shaped structural components [5,6,7].

Despite its numerous advantages, the quality of high-pressure die castings is largely determined by the appropriate selection of process parameters and tool design [8,9]. An improperly designed gating system, unsuitable die cavity filling conditions, or inadequate process parameters can lead to various defects, such as gas and shrinkage porosity, cold shuts, misruns, inclusions, cracks, and air entrainment. Such defects adversely affect the mechanical properties, dimensional accuracy, and reliability of castings and ultimately increase production costs due to additional processing or rejection of defective parts [10,11,12,13]. Therefore, during product development, it is necessary to optimize the casting geometry, gating and venting systems, as well as the filling and solidification parameters, in order to minimize the possibility of defect formation [13,14].

The development of digital technologies in recent years has enabled significant improvements in the design of die castings. One of the methods increasingly being applied is three-dimensional (3D) optical scanning of existing products, which enables the rapid and accurate acquisition of a digital representation of an actual component [15,16]. The resulting point cloud and reconstructed CAD model enable straightforward geometry modification, reverse engineering, and preparation of the model for numerical analyses without the need for extensive measurements or complete remodelling of the product [17,18].

Based on the CAD model thus obtained, numerical simulation of the casting process can be performed to analyze, in a virtual environment, die cavity filling, molten metal solidification, temperature distribution, air entrainment, oxide film formation, porosity, and other critical aspects of the process [8,13,19,20]. The application of simulation tools enables the prediction of potential defects before physical trials and the manufacture of costly production tooling, thereby reducing development costs and the need for repeated tool modifications. The obtained results can subsequently be used to optimize gating and feeding systems and adjust process parameters in order to improve filling and solidification behavior and reduce the likelihood of casting defects [21,22,23,24]. Consequently, the combination of 3D scanning and numerical simulation represents an effective approach to the development and optimization of high-pressure die casting processes.

The aim of this study is to establish a workflow for developing a high-pressure die casting model of an existing differential transmission cover by combining 3D optical scanning, reverse engineering and numerical simulation. The scanned geometry was reconstructed as a CAD model, machining-related features were adjusted where required, and a gating system with overflow elements was designed for subsequent simulation. The numerical analysis was used to examine cavity filling, solidification and predicted indicators of air entrainment and shrinkage porosity before manufacture of production tooling. The contribution is the integration of these steps for the selected component; the simulation results are interpreted as predictions and are not presented as experimentally validated defect measurements.

## 2. Materials and Methods

In this study, a differential transmission cover from a BMW vehicle, manufactured by high-pressure die casting of an aluminum alloy, was selected for the development of a CAD model and numerical analysis of the die casting process. The casting is characterized by a complex geometry with varying wall thicknesses and demanding transitions between individual functional surfaces. The geometry of the existing casting was reconstructed using reverse engineering, resulting in a CAD model suitable for defining the gating system and performing numerical simulation of the die casting process.

**Figure 1:**
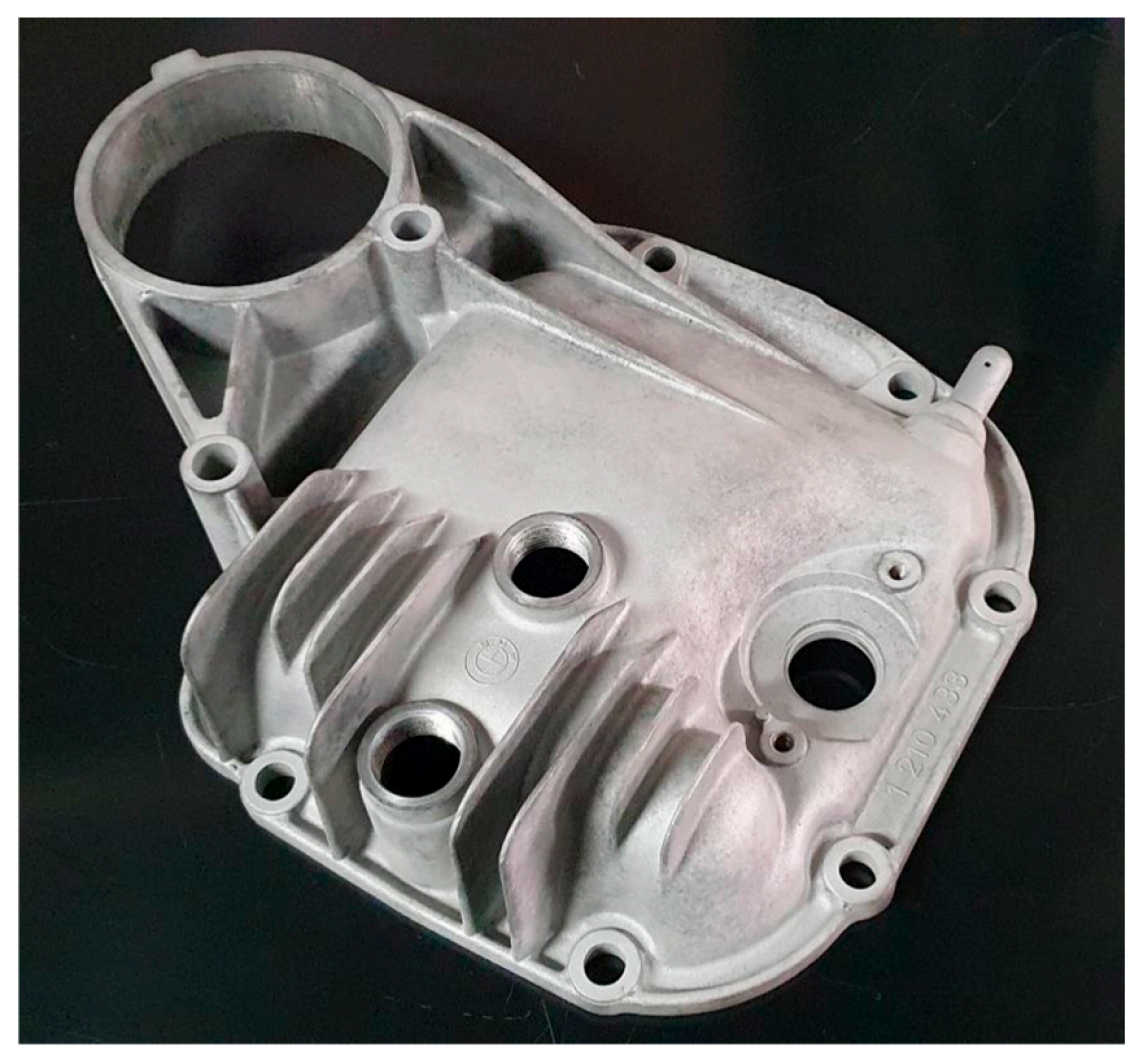
Differential transmission cover after sandblasting, prepared for 3D scanning.

The overall dimensions of the casting were approximately 206 × 104 × 79 mm, while its measured mass was 1.09 kg. Prior to digitization, the casting surface was prepared by sandblasting to remove surface contaminants, oxide layers, and residual material from the manufacturing process. This improved the optical properties of the surface and provided more stable conditions for 3D scanning. The prepared casting is shown in Figure 1.

An ATOS 5X optical 3D scanner (GOM GmbH, Braunschweig, Germany), based on structured blue-light projection, was used for geometric reconstruction. The scanner is equipped with an integrated high-intensity illumination system, enabling measurements over areas of up to 1000 mm while maintaining a short exposure time of 0.2 s per scan.

Prior to measurement, reference points were placed around the casting to enable determination of the spatial position of the component and automatic alignment of individual scans during data processing.

**Figure 2:**
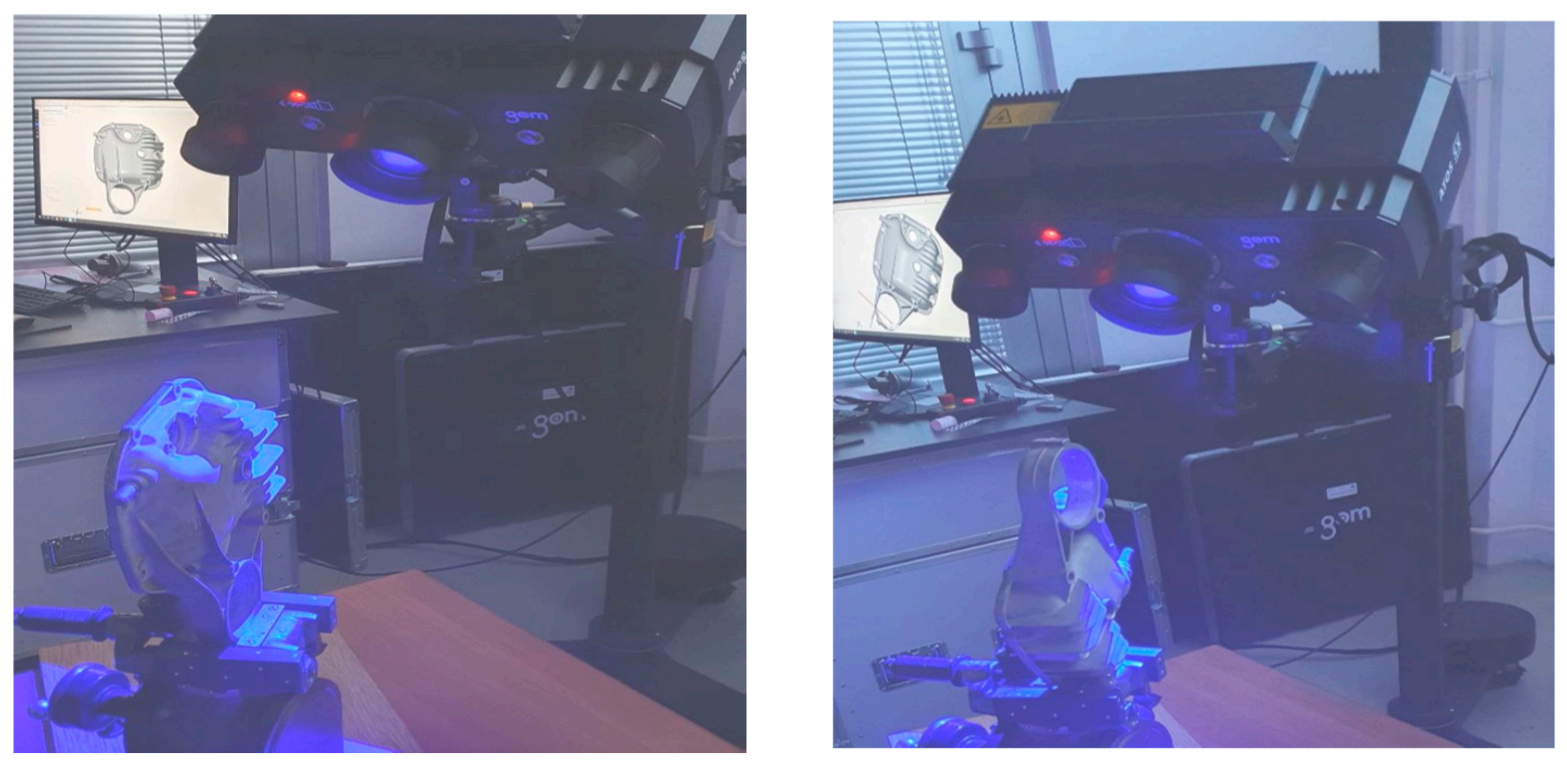
Differential transmission cover secured in a rotary clamping fixture during optical 3D scanning.

Due to the complexity of the geometry and limited accessibility of certain surfaces, scanning was performed in two different clamping positions. In the first position, the casting was placed on the working surface of the scanner. Subsequently, due to insufficient overlap of characteristic geometric features for automatic scan alignment, the casting was additionally secured in a rotary clamping fixture. This arrangement enabled the casting to be gradually rotated and a larger number of surfaces to be scanned while maintaining the relative orientation of the individual scans. An example of the casting setup during the scanning procedure is shown in Figure 2.

After scanning in both clamping positions was completed, the acquired data were processed using the GOM Inspect 2021 software package. The individual point clouds were automatically aligned based on the reference points and geometric characteristics of the surfaces and subsequently merged into a single polygonal model. During processing, measurement noise was removed, the mesh density was optimized, and portions of the geometry that could not be directly captured by optical measurement due to unfavorable surface orientations were reconstructed.

**Figure 3:**
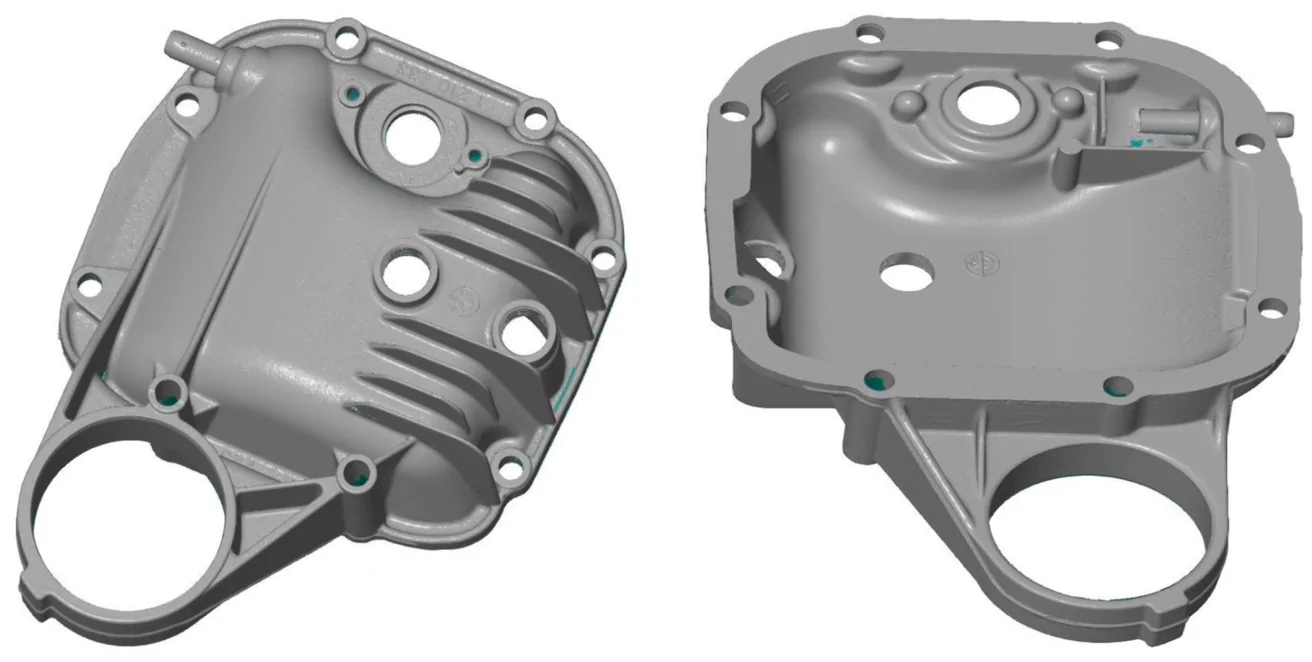
Polygonal model of the differential transmission cover obtained by merging scans from both clamping positions.

Since the original point cloud alone was insufficient for generating the CAD model, additional processing of the surface mesh was performed. Open surfaces generated during scanning were closed to obtain a closed volumetric model, while incompletely defined geometric features were reconstructed based on the surrounding surfaces and structural characteristics of the casting. The final polygonal model obtained by merging the scans of the front and rear sides of the casting is shown in Figure 3.

After all surfaces had been closed and a single polygonal mesh had been generated, the model was exported in an appropriate surface format and further processed in the SolidWorks CAD 2020 software package. Based on the reconstructed polygonal model, a CAD model of the casting was developed using reverse engineering. During this process, the geometry was simplified, details with no significant influence on the casting process were removed, and the surfaces required to generate a closed solid model were defined. Appropriate machining allowances for subsequent machining after casting were also incorporated into the reconstructed geometry.

**Figure 4:**
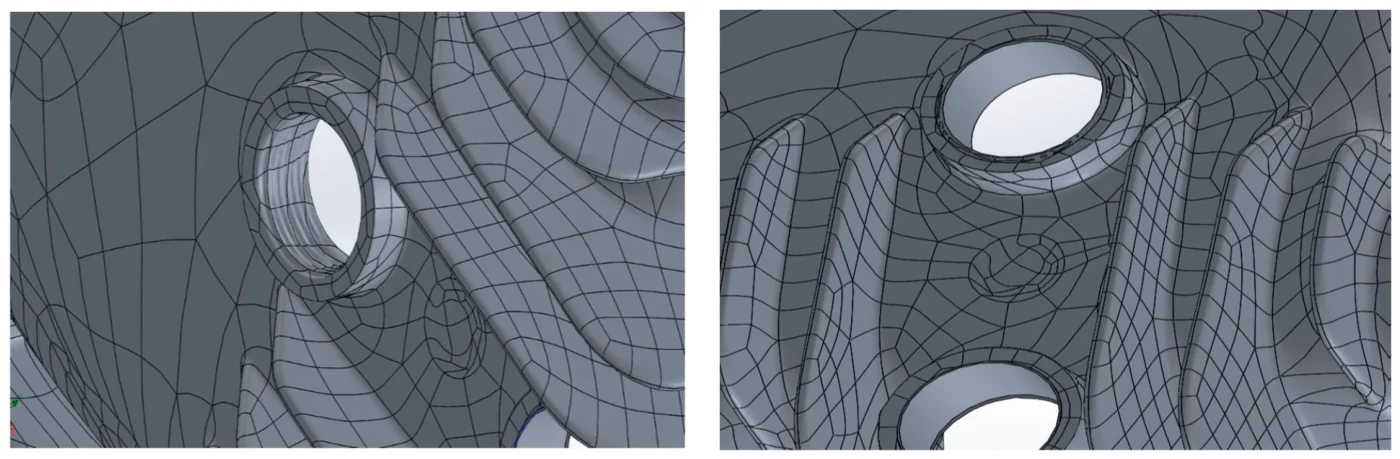
Adjustment of the diameters of bores intended for subsequent thread machining.

Particular attention was given to the bores intended for subsequent thread machining. Since the final geometry of the threaded bores is obtained by subsequent machining, their initial diameters were adjusted to ensure that a sufficient amount of material remained after casting for finishing operations and achievement of the required final dimensions. The adjustment of the bore diameters is shown in Figure 4.

**Figure 5:**
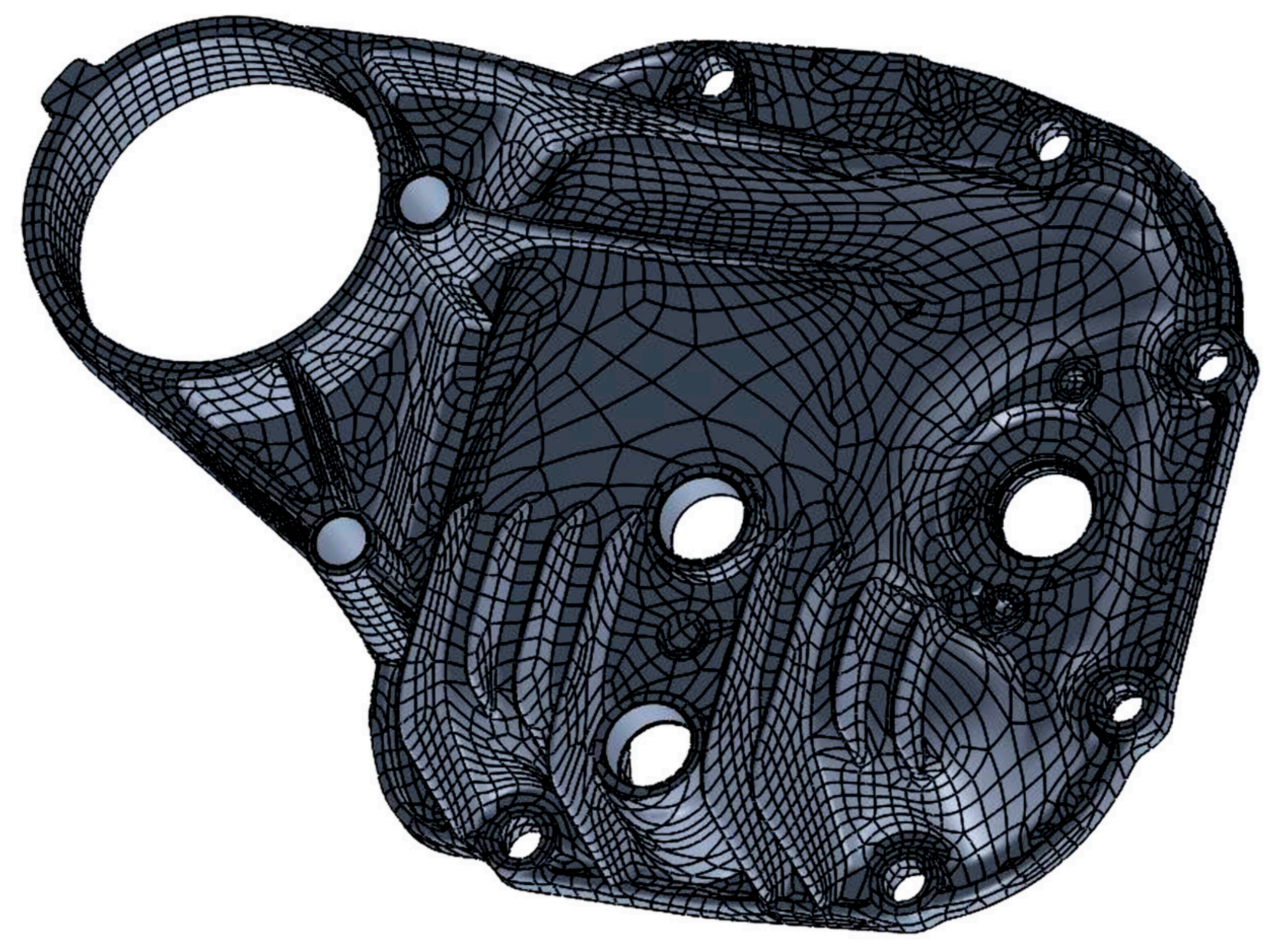
Final CAD model of the differential transmission cover developed using reverse engineering in SolidWorks.

The final CAD model of the differential transmission cover was obtained using reverse engineering and served as the basis for defining the gating system and preparing the numerical simulation of the die casting process. The final CAD model developed in the SolidWorks software package is shown in Figure 5.

**Figure 6:**
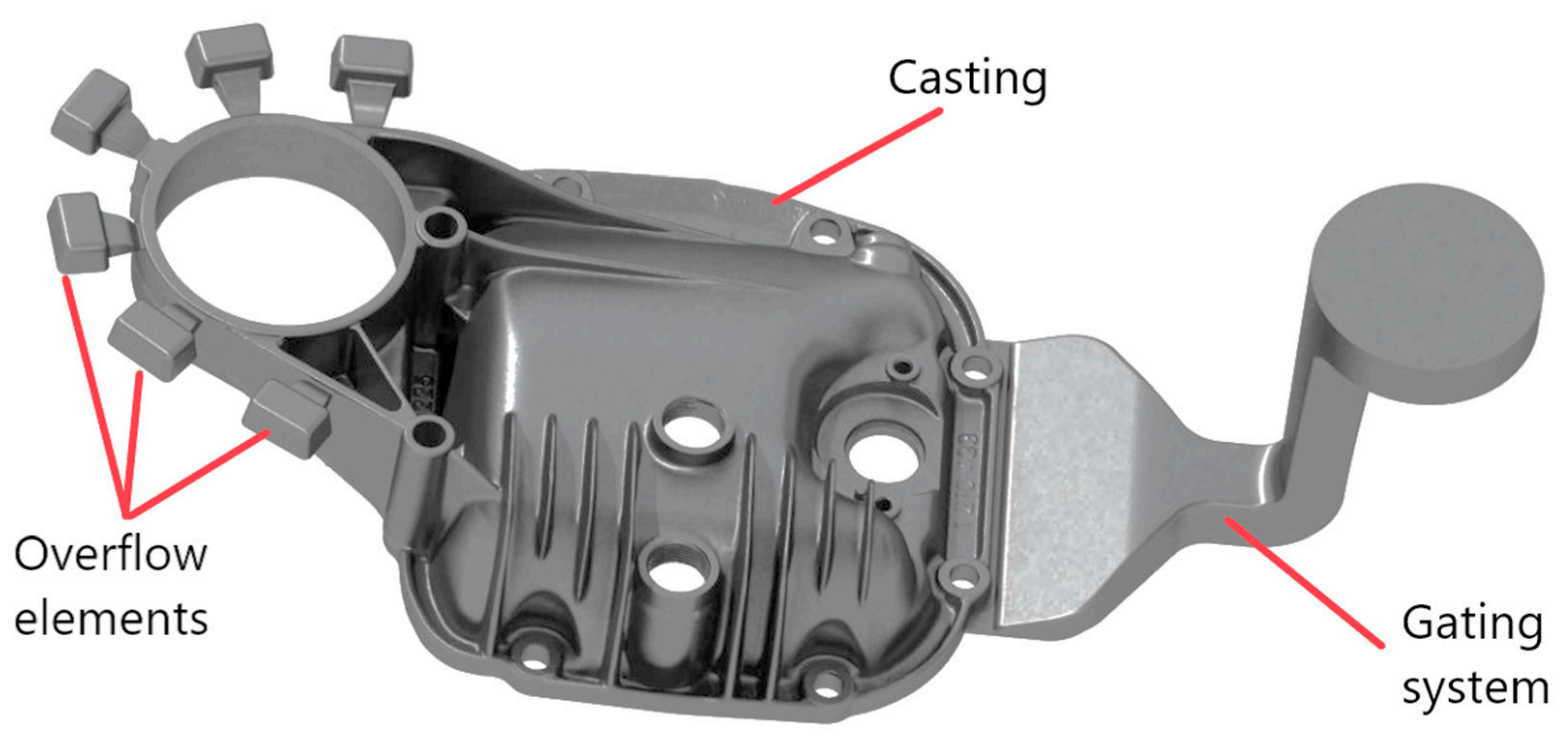
CAD model of the differential transmission cover with the gating system and overflow elements prepared for numerical simulation of the die casting process.

After defining the casting geometry, the gating system was developed, including the runners and venting/overflow elements. In this way, a CAD model of the casting with the associated gating system elements was obtained, which served as the basis for preparing the numerical simulation of the die casting process. The model of the differential transmission cover with the gating system and overflow elements is shown in Figure 6.

**Figure 7:**
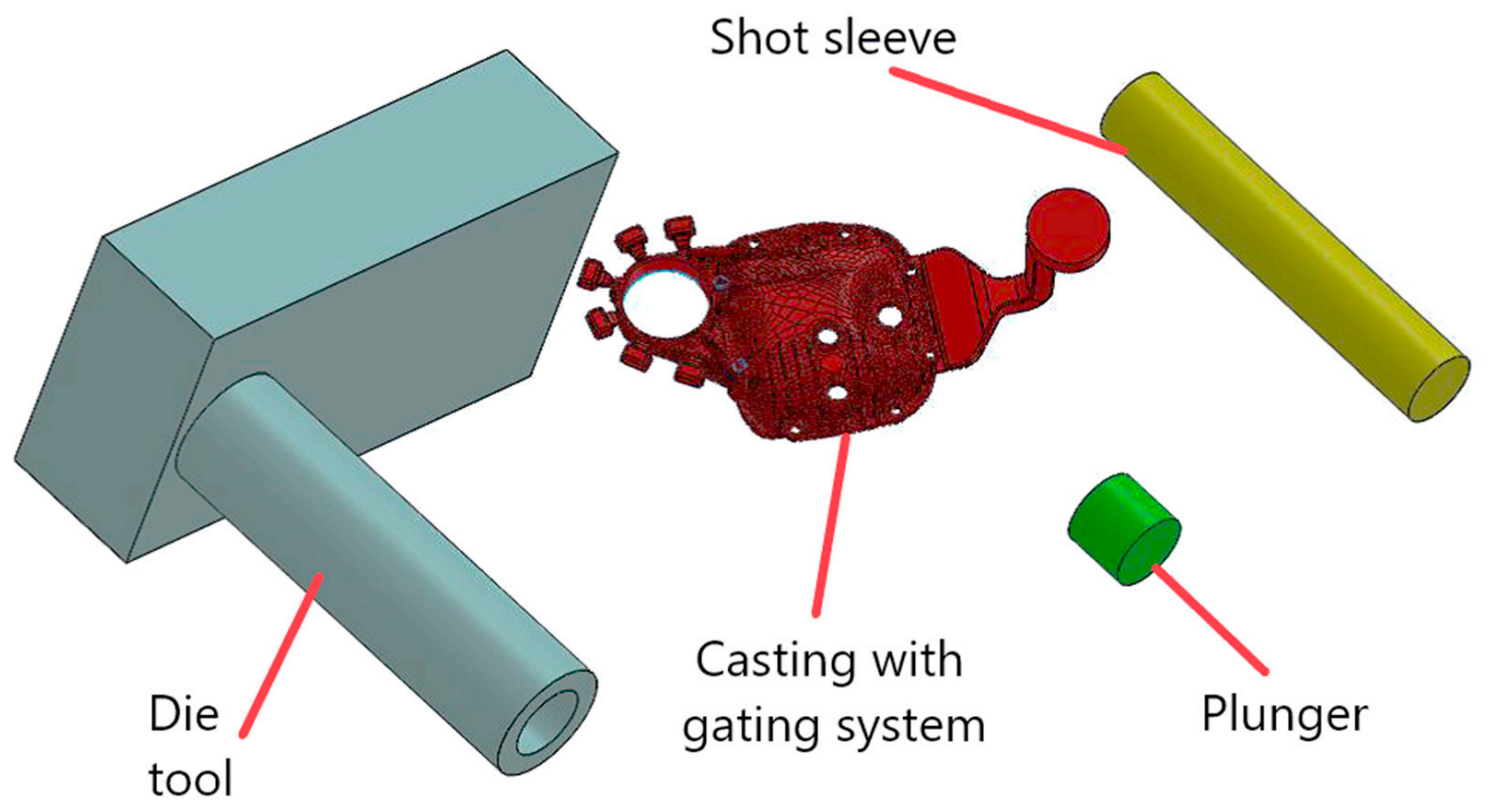
Complete numerical model of the die casting process with the defined die tool, shot sleeve, plunger, and casting model with the gating system.

For the simulation in the ProCAST (Ver. 2023.0) software package, all additional elements required to describe the die casting process were defined based on the existing model. The numerical model was extended by the die tool, shot sleeve, and plunger, thereby forming the complete geometric basis of the casting process. All elements of the numerical model, including the casting with the gating system, die tool, shot sleeve, and plunger, are shown in Figure 7.

**Figure 8:**
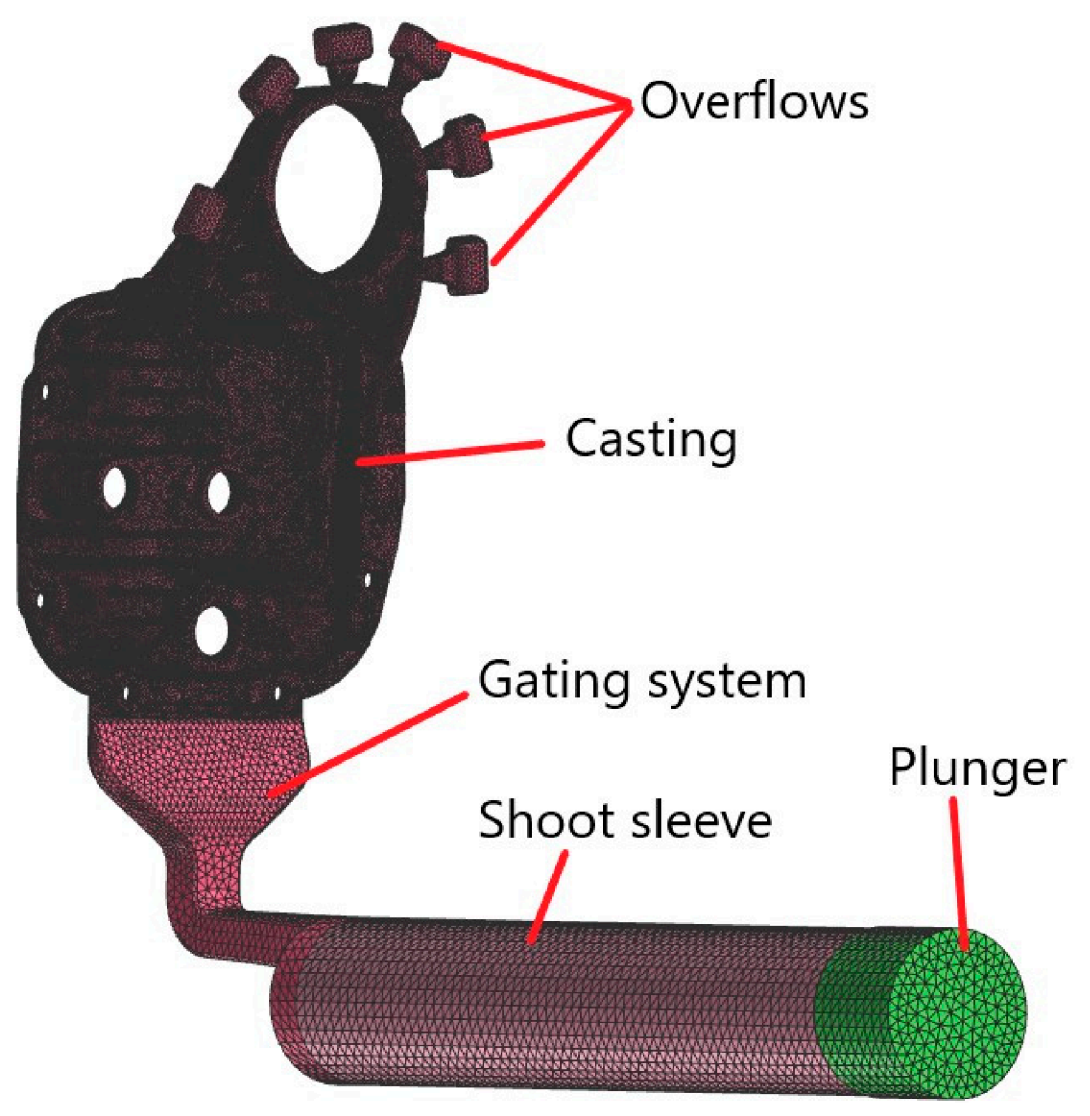
Distribution of the computational mesh in the numerical model.

The numerical model of the high-pressure die casting (HPDC) process was established in ProCAST. The computational mesh was defined to represent the casting geometry with sufficient spatial resolution while maintaining reasonable computational efficiency. The final mesh consisted of 149,344 2D and 2,699,384 3D elements. The mesh was locally refined in the casting region to provide a more detailed representation of the calculated fields while limiting the overall computational cost. The distribution of the computational mesh is presented in Figure 8.

The material properties were obtained from the ProCAST material database. The casting alloy was AlSi_9_Cu_3_, with a liquidus temperature of 586 °C and a solidus temperature of 510 °C. Its thermal conductivity was 140 W/(m · K) at room temperature and 85 W/(m · K) at the pouring temperature of 680 °C. CuCoBe (beryllium copper) was assigned to the plunger, whereas H13 tool steel was used for the metal mould. The initial temperatures of the plunger and metal mould were set to 80 °C and 210 °C, respectively, while the initial temperature of the molten alloy was set to 680 °C. The materials and initial temperatures assigned to the individual components are summarized in Table 1.

The metal mould was coated with Spuncote 10C, a water-based coating, with a nominal thickness of 100 µm. Additional cooling of the metal mould was not explicitly simulated; instead, air at 20 °C was specified as the cooling medium. Heat transfer between the molten metal and the metal mould, as well as between the molten metal and the plunger, was defined using heat transfer coefficients (HTC). The corresponding values assigned to these interfaces are listed in Table 2.

**Figure 9:**
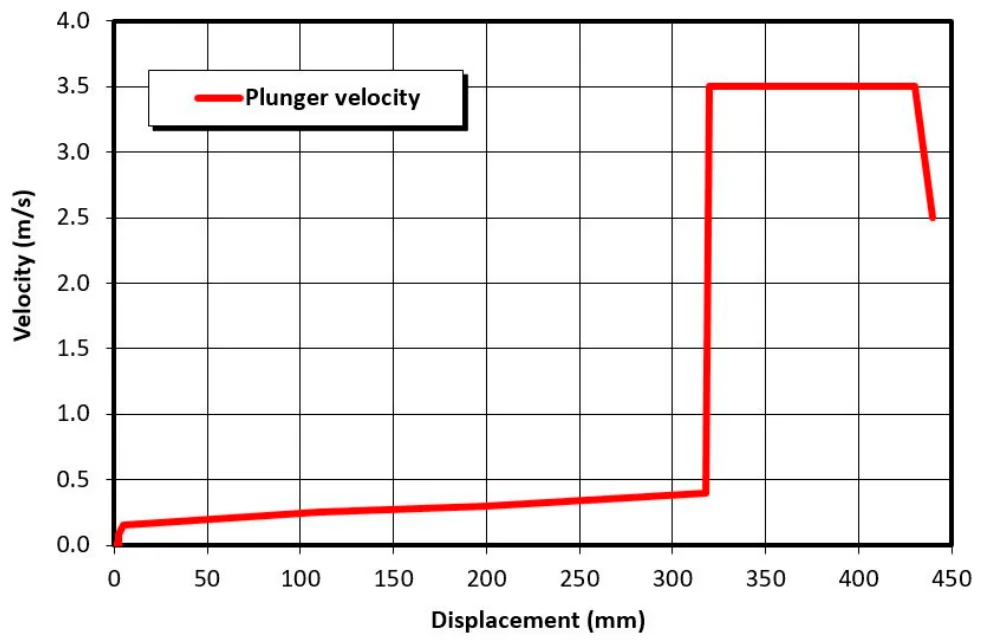
Prescribed plunger velocity as a function of displacement during the HPDC process.

The plunger motion was defined in three successive phases based on the prescribed relationship between plunger displacement and velocity, as shown in Figure 9. The first phase, corresponding to the slow-shot stage, extended from the initial plunger position to a displacement of 320 mm. During this phase, the plunger velocity increased approximately linearly, reaching 0.4 m/s. The second phase, corresponding to the fast-shot stage, covered the displacement range from 320 to 430 mm, during which the plunger velocity increased to 3.5 m/s. The third phase extended from 430 to 440 mm and represented the final stage of plunger movement, associated with pressure intensification in the HPDC process. This phase covered the shortest displacement interval and was characterized by a reduction in plunger velocity from approximately 3.5 to 2.5 m/s. Although pressure was not prescribed directly in the numerical model, the final plunger movement represented the stage associated with pressure intensification during the casting process.

The numerical simulation was subsequently used to investigate cavity filling, solidification, and temperature evolution during the casting process. The results were also analysed to identify potential casting defects, including air entrainment and shrinkage porosity, as well as regions exhibiting unfavourable solidification conditions.

## 3. Mathematical Model

The numerical simulation of the high-pressure die casting (HPDC) process was performed using the ProCAST software. The mathematical model describes the coupled phenomena of molten metal flow, heat transfer, solidification, and free-surface evolution during mold filling. The governing equations are based on the conservation of mass, momentum, and energy. Turbulent flow is described using the standard κ − ε turbulence model, while the free surface of the molten metal is tracked using the Volume of Fluid (VOF) method [25,26,27].

The conservation of mass is described by the continuity Equation (1):

(1) $$\frac{\partial \rho }{\partial t}+\frac{\partial (\rho u_{i})}{\partial x_{i}}=0\;$$

where *ρ* is the density, *t* is time, and *u_i_* represents the velocity component in the *x_i_* coordinate direction.

The motion of the molten metal is described by the momentum conservation equation. In the presence of a solidifying region, the momentum equation accounts for the resistance to flow in the mushy zone (2):

(2) $$\rho \frac{\partial u_{i}}{\partial t}+\rho u_{j}\frac{\partial u_{i}}{\partial x_{j}}+\frac{\partial }{\partial x_{j}}\left(p\delta_{\mathrm{ij}}(µ+{µ}_{T})\frac{\partial u_{i}}{\partial x_{j}}\right)=\rho g_{i}-\frac{µ}{K}u_{i}$$

where *p* is pressure, *δ_ij_* is the Kronecker delta, µ is the dynamic viscosity, µ*_T_* is the turbulent viscosity, *g_i_* is the gravitational acceleration component, and *K* is the permeability of the mushy zone.

The permeability of the solidifying region is calculated using the Kozeny–Carman relationship (3):

(3) $$K=\;\frac{f_{l}^{3}}{5{\cdot M}_{s}^{2}{\left(1-f_{l}\right)}^{2}}$$

where *f_l_* is the liquid fraction and *M_s_* is a microstructural parameter defined as (4):

(4) $$M_{s}=\frac{6\lambda }{D_{p}}$$

where *λ* represents the characteristic microstructural spacing and *D_p_* the characteristic particle or dendrite size.

Heat transfer during mold filling and solidification is described using an enthalpy formulation. The transient energy equation for molten metal flow is given by (5):

(5) $$\rho \frac{\partial H}{\partial T}\frac{\partial T}{\partial t}-\nabla \left[k\;\nabla \;T\right]-q\left(x\right)=0$$

where *H* is the specific enthalpy, *k* is the thermal conductivity, *T* is temperature, and *q* represents a volumetric heat source.

For turbulent flow, the effective thermal conductivity includes the contribution of turbulent heat transport (6):

(6) $$\rho \frac{\partial H}{\partial t}+\rho u_{i}\frac{\partial H}{\partial x_{i}}-\nabla \left[\left(k+\frac{{µ}_{T}}{\sigma_{T}}\right)\nabla T\right]-q=0$$

where *σ_T_* is the turbulent Prandtl number.

The specific enthalpy is defined as (7):

(7) $$H\left(T\right)=\;\int_{0}^{T}c_{p}\;d_{T}+L_{T}[1-f_{s}\left(T\right)]$$

where *c_p_* is the specific heat capacity, *L_T_* is the latent heat of solidification, and *f_s_* is the solid fraction. The enthalpy formulation therefore accounts for both sensible and latent heat during the solidification process.

The turbulent flow of the molten metal is described using the standard κ − ε model. The transport equation for turbulent kinetic energy is (8):

(8) $$\frac{\partial (\rho \kappa )}{\partial t}+u_{j}\frac{\partial (\rho \kappa )}{\partial x_{j}}-\frac{\partial }{\partial x_{j}}\left(\frac{{µ}_{T}}{\sigma_{\kappa }}\frac{\partial \kappa }{\partial x_{j}}\right)-{µ}_{T}G_{T}-\rho \varepsilon$$

The corresponding transport equation for the dissipation rate is (9):

(9) $$\frac{\partial (\rho \varepsilon )}{\partial t}+u_{j}\frac{\partial (\rho \varepsilon )}{\partial x_{j}}-\frac{\partial }{\partial x_{j}}\left(\frac{{µ}_{T}}{\sigma_{\varepsilon }}\frac{\partial \varepsilon }{\partial x_{j}}\right)-\frac{\varepsilon }{\kappa }(C_{1}{µ}_{T}G_{T}-C_{2}\rho \varepsilon )$$

The turbulent viscosity is determined from the turbulent kinetic energy and its dissipation rate according to (10):

(10) $${µ}_{T}=\frac{C_{µ}\rho \kappa^{2}}{\varepsilon }$$

where *G_T_* is the production term of turbulent kinetic energy, while *Cμ*, *C*_1_, and *C*_2_ are model constants. For the standard κ − ε model, the commonly used values are the commonly used values of these constants are presented in Table 3:

The solidification process is coupled with the thermal model through the solid fraction and latent heat release. At the grain scale, the thermal balance is described by (11):

(11) $$\left(\rho c_{p}\frac{\mathrm{dT}}{\mathrm{dt}}-\rho L_{T}\frac{df_{s}}{\mathrm{dt}}\right)\cdot \frac{4}{3}\pi \cdot r_{I}^{3}=Q_{\mathrm{ext}}\cdot 4\pi r_{I}^{2}$$

where *r_l_* is the grain radius and *Q_ext_* is the external heat flux acting on the solidifying grain.

The evolution of the solid fraction is described using the Johnson–Mehl approximation. The change in solid fraction with time is given by (12):

(12) $$\frac{\partial f_{s}}{\partial t}=\left(1-f_{s}\right)\cdot 4\pi \cdot r_{e}^{2}N\frac{dr_{e}}{\mathrm{dt}}$$

where *r_e_* is the radius of the eutectic grain and *N* is the number of nucleation sites. The factor 1 − *f_s_* accounts for the impingement of growing grains during the later stages of solidification.

## 4. Results and Discussions

The numerical simulation of the die casting process was performed using the finite element method in the ProCAST software package. Prior to the simulation, all process parameters required to describe the casting process were defined, including material properties, molten metal temperature, plunger velocity, injection pressure, heat transfer conditions, and other boundary conditions. Based on the defined numerical model, the filling of the die cavity, molten metal solidification, and the potential occurrence of characteristic casting defects were analyzed.

One of the important parameters in the die casting process is the plunger velocity, which determines the different stages of the injection process. The plunger movement was divided into a slow shot phase, a fast shot phase, and a pressure holding phase. During the slow shot phase, the plunger velocity must be sufficiently controlled to ensure a gradual increase in the molten metal level in the shot sleeve and to prevent excessive surface wave formation. During the fast shot phase, the plunger velocity is significantly increased to enable rapid filling of the gating system and die cavity. An excessively high or improperly selected velocity during this phase may cause pronounced turbulence and surface wave formation, increasing the possibility of air entrapment in the molten metal.

**Figure 10:**
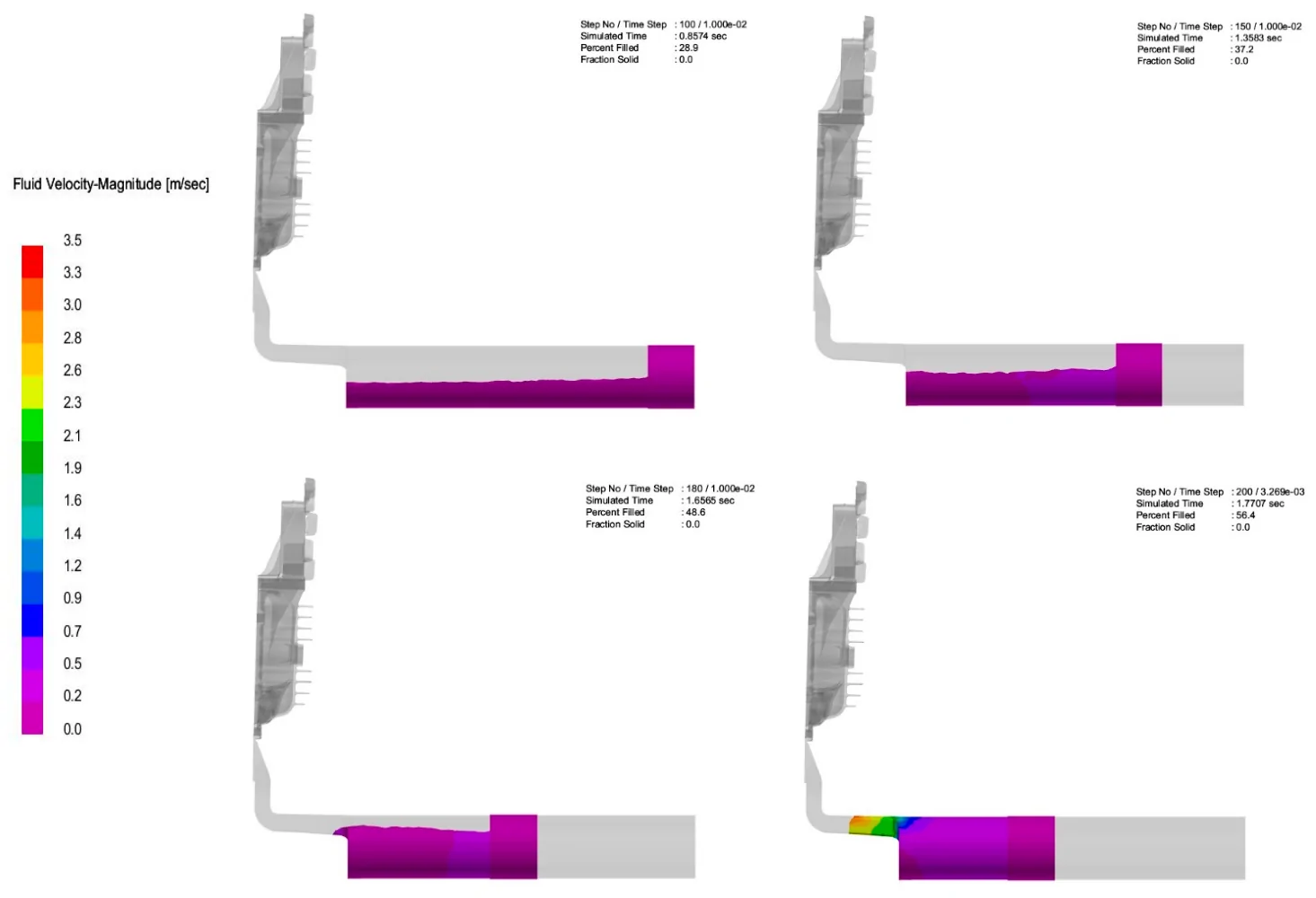
Simulated metal flow velocity distribution at selected time steps during the die casting process.

The variation in plunger velocity during the die casting process is shown in Figure 10. During the slow shot phase, the molten metal level gradually increases without pronounced turbulence or sudden separation of the surface wave, indicating appropriately selected plunger velocity and acceleration. After the molten metal enters the gating system, the fast shot phase begins, during which the increased plunger velocity enables rapid filling of the die cavity. After the die cavity has been filled, the pressure holding phase follows, during which pressure is maintained on the molten metal during the initial stage of solidification.

**Figure 11:**
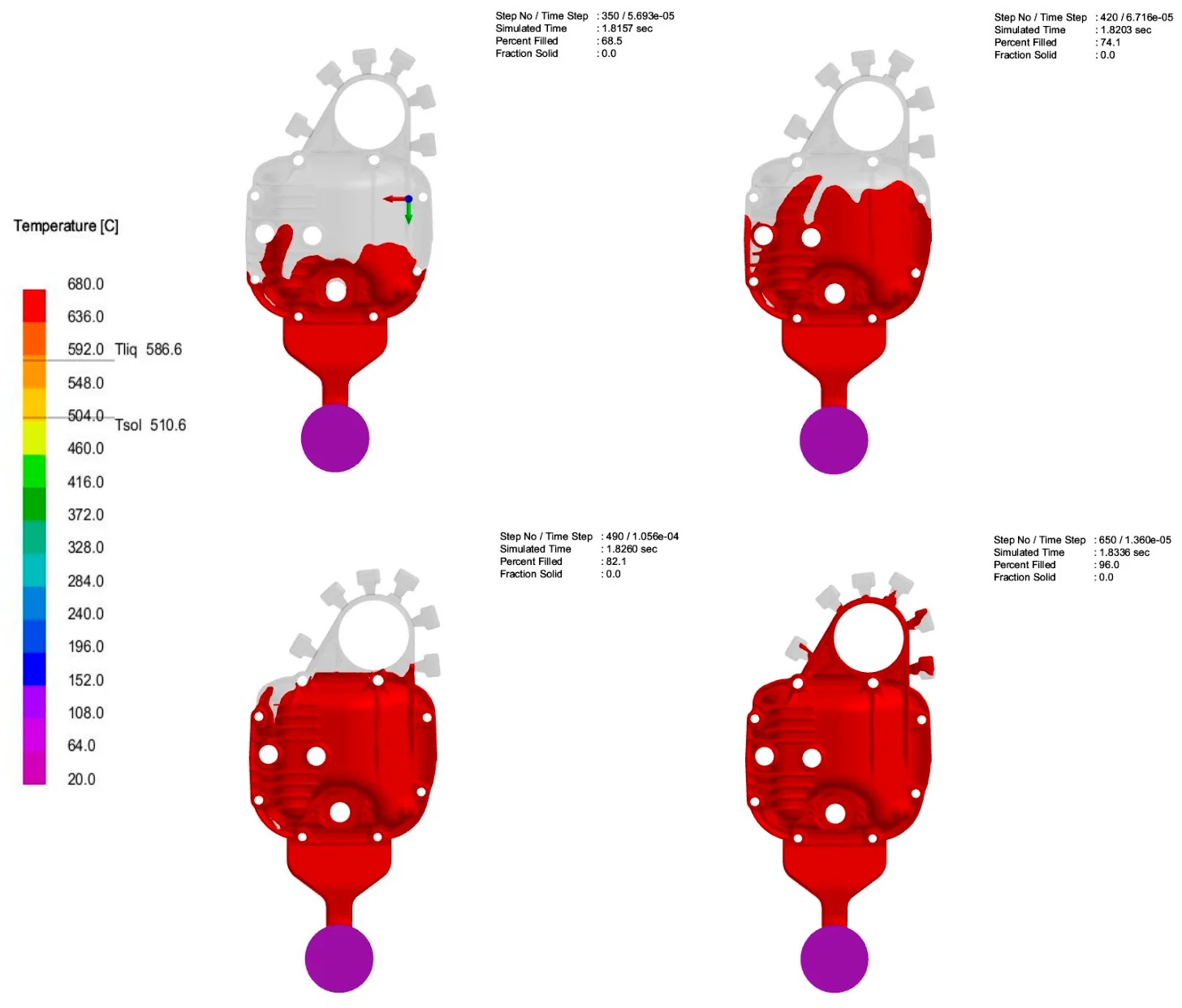
Filling of the mould cavity during the die casting process.

The results of the mould cavity filling simulation are shown in Figure 11. The molten metal is distributed uniformly through the gating system and gradually fills the casting sections without pronounced turbulence. The overflow elements are observed to be filled among the last regions, which corresponds to their position relative to the direction of molten metal flow and enables the molten metal from the final filling regions to be received. At the same time, the simulation shows that the mould cavity is completely filled before the molten metal temperature falls below the liquidus temperature. This reduces the possibility of cold shuts and misruns, i.e., incomplete filling of the casting.

**Figure 12:**
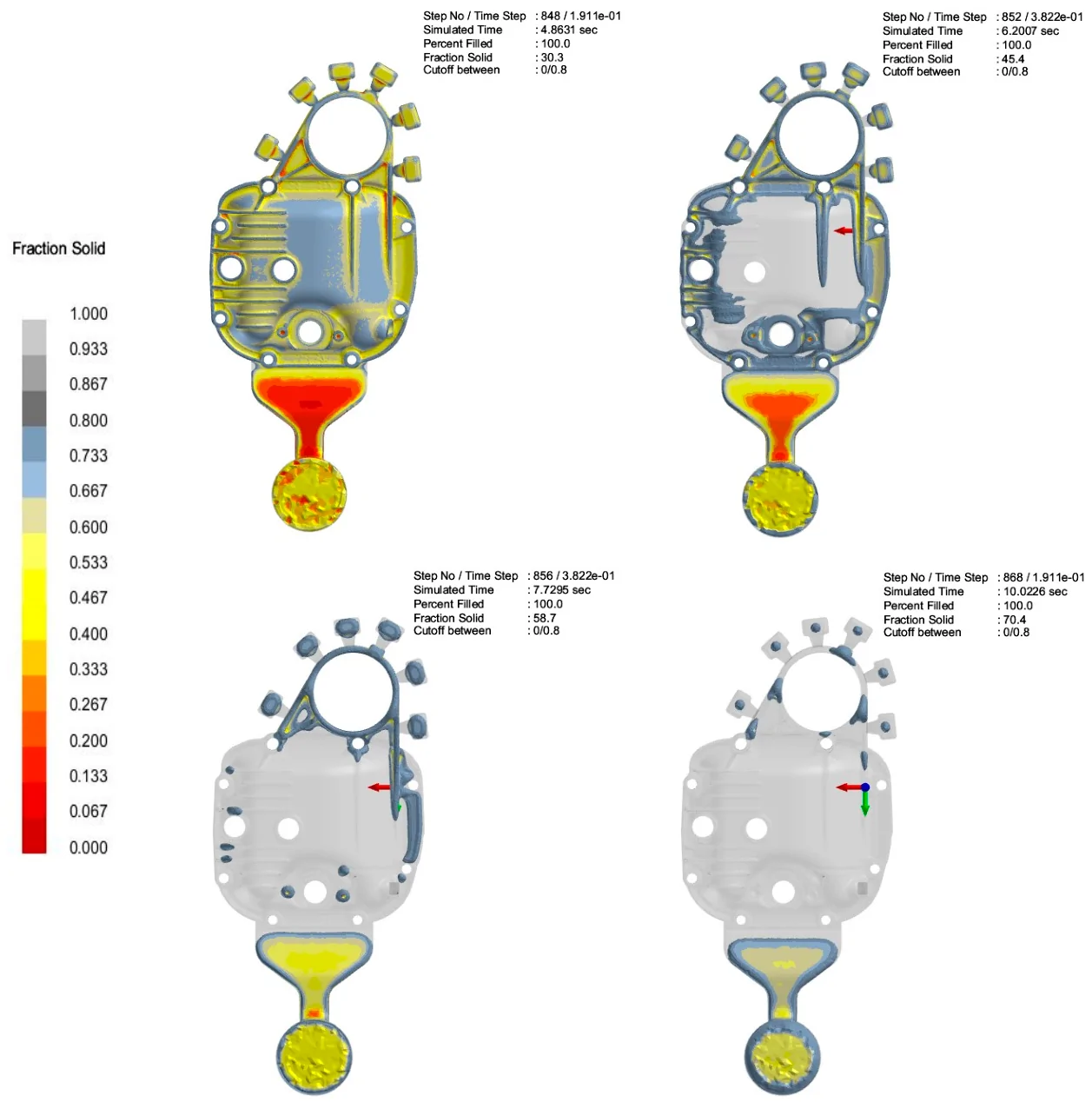
Distribution of the solid fraction during solidification of the casting.

After completion of the filling phase, the distribution of the solid fraction was analyzed. The results shown in Figure 12 indicate that local thickened sections of the casting, particularly the areas around the bore reinforcements and larger ribs, are the last to solidify. Such areas represent potential zones for shrinkage porosity due to material contraction during solidification. Their identification is therefore important for optimizing the casting geometry and the positions of the gating system and overflow elements.

**Figure 13:**
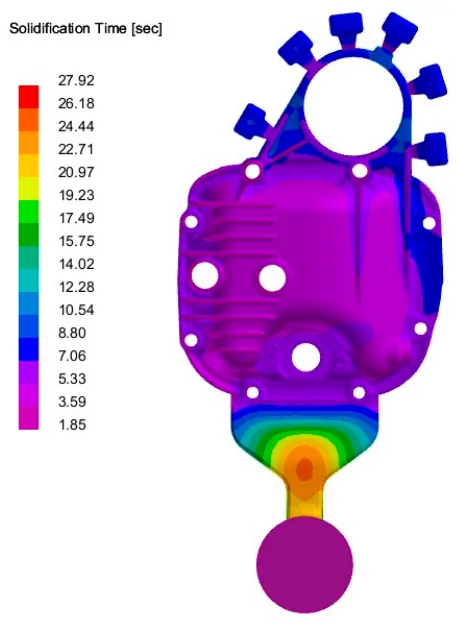
Distribution of solidification time in the casting.

For a better assessment of the solidification dynamics, the time required for the molten metal to transition from the liquidus temperature of 586.6 °C to the solidus temperature of 510.6 °C was determined. The distribution of solidification time is shown in Figure 13, with the solidification time of the casting ranging from 1.85 to 8.80 s. The results show that the regions near the overflow elements and certain local areas with greater wall thickness are the last to solidify. This result is expected due to the slower heat removal from the more massive sections of the casting. Properly positioned overflow elements enable the excess molten metal and air from the final filling regions to be received and may contribute to reducing the possibility of shrinkage porosity during solidification.

**Figure 14:**
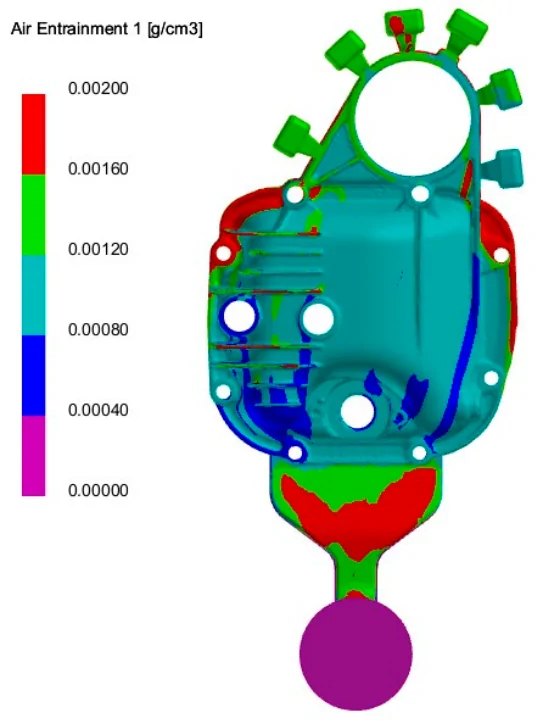
Distribution of air entrainment in the casting.

One of the characteristic defects that may occur during die casting is air entrapment during filling of the die cavity. The simulation results shown in Figure 14 indicate that the highest predicted air entrainment is located in the final filling regions and near certain cooling ribs. The values of the air-entrainment indicator in the analyzed regions range from 0.0004 to 0.002 g/cm^3^. These values represent numerical simulation outputs and are reported descriptively. Without an independently established acceptance threshold or experimental validation, they cannot be used to conclude that the predicted air entrainment is negligible or that significant gas porosity will not occur. Nevertheless, the spatial distribution of the predicted air entrainment provides insight into the regions where air entrapment is most likely to occur during die cavity filling.

**Figure 15:**
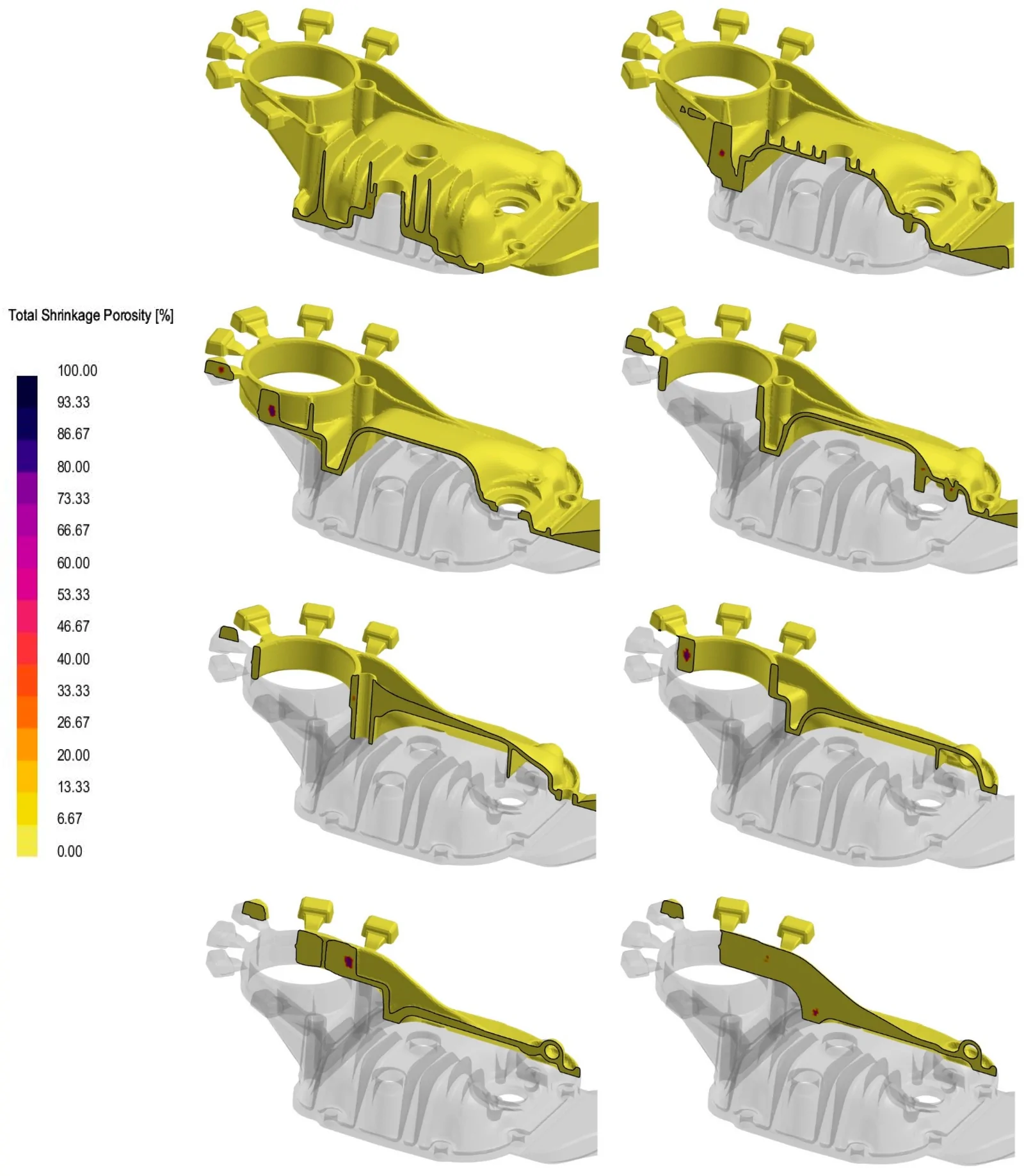
Distribution of predicted total shrinkage porosity in different views and cross-sections of the casting.

Based on the numerical simulation results, the distribution of the predicted shrinkage-related indicator was analysed. Examination of multiple views and cross-sections enabled a more detailed assessment of the regions potentially susceptible to shrinkage porosity, as shown in Figure 15. The results revealed a non-uniform distribution of predicted total shrinkage porosity throughout the casting, with localized areas exhibiting elevated values.

The highest predicted value, approximately 80%, was identified in the region surrounding the upper mounting hole of the differential housing cover, indicating that this region is potentially the most susceptible to shrinkage porosity. The elevated value may be associated with the local geometry and the resulting solidification conditions. Variations in wall thickness and adjacent geometrical transitions can influence heat extraction and the progression of solidification. If the surrounding material solidifies earlier, the supply of liquid metal required to compensate for volumetric contraction may become restricted, increasing the susceptibility to shrinkage porosity in this region. Porosity in this area could reduce the effective load-bearing cross-section and adversely affect the mechanical performance of the component, particularly under mechanical and dynamic loading.

Overall, the simulations provide an initial assessment of the regions potentially susceptible to shrinkage porosity and can support the identification of areas requiring further investigation during die-casting process development [13,20,28]. The predicted values should be regarded as numerical estimates of shrinkage porosity rather than definitive measurements of the actual defect content. Their reliability must therefore be evaluated through experimental validation, involving the inspection of production castings and comparison of the observed defects with the predicted locations [13,19,20,29]. Such validation would help establish the extent to which the simulation results represent actual casting conditions. Despite this limitation, the integration of 3D scanning, reverse engineering and numerical simulation provides a useful approach for identifying potential casting defects and supporting process development before production tooling is manufactured.

## 5. Conclusions

The high-pressure die casting process of a differential transmission cover was analyzed using a reconstructed CAD model and numerical simulation. The applied 3D scanning and reverse engineering approach enabled the development of a digital model of the existing casting, which was used as the basis for defining the gating system and performing the numerical simulation of the casting process.

The numerical simulation results showed that the defined plunger movement parameters enabled stable filling of the shot sleeve and die cavity without pronounced turbulence. The die cavity was completely filled before the molten metal temperature dropped below the liquidus temperature, thereby reducing the possibility of cold shuts and misruns. The solidification analysis showed that the last-solidifying regions were mainly located in local thickened sections of the casting and near the overflow elements, identifying these regions as potentially critical areas for shrinkage porosity formation.

The air entrapment analysis showed that the largest amount of entrapped air occurred in the final filling regions; however, the total predicted amount was small and localized. The analysis also showed that the regions with the highest probability of shrinkage porosity formation generally corresponded to the areas of last solidification, particularly in local thickened sections of the casting. An increased probability of shrinkage porosity formation was also observed in the regions immediately upstream of the overflow elements, indicating the need for their further optimization to reduce the possibility of shrinkage porosity formation.

The obtained results indicate that the defined gating and overflow system provides favorable filling and solidification conditions for the analyzed casting. At the same time, regions requiring particular attention during further process optimization were identified, demonstrating the usefulness of numerical simulation for the timely identification of potential casting defects prior to the manufacture of the production tooling. The combination of 3D scanning, reverse engineering, and numerical simulation therefore represents a suitable approach for the development and optimization of high-pressure die casting processes and may contribute to reducing the number of tooling modifications, development time, and production costs.

## Figures and Tables

**Table 1:** Materials and initial temperatures assigned to the components of the HPDC simulation.

Component	Materials	Initial Temperature
Plunger	CuCoBe	80 °C
Metal mould	Steel H13	210 °C
Molten metal	AlSi_9_Cu_3_	680 °C

**Table 2:** The amount of HTC at contacts of different interfaces.

Interfaces in Contacts	HTC (W/m^2^K)
Molten metal/Metal mould	1700–2000
Molten metal/Plunger	3500

**Table 3:** Model constants used in the standard *κ* − *ε* turbulence model.

*Cμ*	*C*_1_	*C*_2_
0.09	1.44	1.92

## Data Availability

The original contributions presented in the study are included in the article, further inquiries can be directed to the corresponding author.
